# Immune checkpoint inhibitor restores daily function in patient with microsatellite instability (MSI)-high advanced endometrial cancer and poor performance status

**DOI:** 10.1007/s13691-025-00752-3

**Published:** 2025-02-14

**Authors:** Ayaka Matsui, Taichi Yoshida, Yuya Takahashi, Koji Fukuda, Kazuhiro Shimazu, Daiki Taguchi, Hanae Shinozaki, Naoaki Kodama, Shunsuke Kato, Hironori Waki, Hiroshi Nanjo, Hiroyuki Shibata

**Affiliations:** 1 Department of Clinical Oncology, Graduate School of Medicine, Akita University, Akita, Japan; 2 Department of Metabolism and Endocrinology, Graduate School of Medicine, Akita University, Akita, Japan; 3 Department of Pathology, Akita University Hospital, Akita, Japan

**Keywords:** Performance status, Microsatellite instability-high, Endometrial cancer, Immune checkpoint inhibitor, Pembrolizumab

## Abstract

The immune checkpoint system suppresses T-cell activity. Unlike cytotoxic anticancer drugs that directly kill cells, immune checkpoint inhibitors (ICIs) are generally safer by stimulating tumor immunity. However, most clinical trials require patients to have a better performance status (PS), leaving limited evidence for those with poorer PS. In practice, patients may be classified with poor PS due to tumor-induced pain and motor dysfunction, even if major organs remain functional. Real-world data on non-small cell lung cancer has shown no safety difference between patients with PS 3/4 and those with lower PS. Approximately 20–30% of endometrial cancer cases show microsatellite instability-high (MSI-high), the highest among common malignancies. A 46-year-old patient with advanced, recurrent endometrial cancer resistant to standard chemotherapy, and PS of 4 from severe pelvic pain, was diagnosed with MSI-high. Pembrolizumab was initiated and continued for 19 courses, after which lesions had disappeared or calcified, leading to drug discontinuation. Now, 4 and a half years post-treatment, she has regained independent mobility and returned to work, and her PS has improved to approximately 1. Side effects included Grade 2 or lower thyroiditis, hypothyroidism, and hypoadrenalism, manageable with hormone replacement therapy and temporary pembrolizumab suspension. This case underscores the need to test for MSI-high/mismatch repair deficiency in endometrial cancer and to consider ICI therapy in patients with poor PS but no major organ dysfunction. In such cases, ICI can rapidly improve overall condition, a phenomenon known as a Lazarus-type response, as seen in other cancers such as non-small cell lung cancer.

**Supplementary Information:**

The online version contains supplementary material available at 10.1007/s13691-025-00752-3.

## Introduction

Many cancer chemotherapy clinical trials set performance status (PS) as part of their eligibility criteria, typically allowing patients with an Eastern Cooperative Oncology Group (ECOG) PS of 0, 1, or 2 [1]. However, in clinical practice, Stage IV patients with a PS of 3 or 4 are often encountered. PS can be somewhat subjective and may vary based on the evaluator’s assessment. Additionally, when activities of daily living (ADL) are impaired due to cancer invasion or metastasis, particularly in the musculoskeletal system, such as with bone metastases, the PS declines [2]. This apparent PS observed in practice can hinder patients from receiving appropriate drug therapy. Kawano et al. referred to these conditions as cancer locomotive syndrome and raised concerns about this issue [3].

Furthermore, recent advancements in drug therapy have led to the introduction of various molecular targeted drugs (MTDs) and immune checkpoint inhibitors (ICIs) into clinical practice. For cytotoxic anticancer drugs, administration is generally not advised for patients with poor PS due to concerns about adverse events [1]. In contrast, MTDs and ICIs tend to have fewer adverse events compared to cytotoxic agents [4]. Consequently, MTDs and ICIs may be considered for patients with a PS of 3 or 4. For instance, in non-small lung cell cancer (NSCLC), patients with an *epidermal growth factor receptor* (*EGFR*) gene mutation or *ALK* gene fusion may receive MTDs [5]. Lung cancer guidelines suggest molecular targeted therapy for patients with PS 3 or 4 who have *EGFR* or *ALK* variants, albeit weakly [6]. The use of ICIs, however, remains a topic of debate [1].

This report discusses a case of endometrial cancer with a PS of 4. Managing unresectable advanced endometrial cancer requires a personalized treatment approach that may include surgery, chemotherapy, radiation, or hormone therapy, depending on the patient’s specific circumstances.

The frequency of microsatellite instability (MSI) is highest in endometrial cancer, estimated at about 17% [7]. In the KEYNOTE-158 study, pembrolizumab (Pem) demonstrated durable antitumor effects and favorable survival rates with manageable toxicity in patients with advanced MSI-high/mismatch repair-deficient (dMMR) endometrial cancer who had prior treatments and a PS of 0–1 [7].

A patient with advanced endometrial cancer and a PS of 4 came to our department. Her previous physician recommended palliative care, believing that further drug therapy was not feasible. However, the patient was eager for treatment, prompting her referral to our department. MSI testing had not yet been conducted, so it was performed and identified as MSI-high. We assessed that the poor PS of 4 resulted from cancer-related pain and concluded that ICI administration was appropriate. Following ICI treatment, her ADL improved rapidly, allowing her to walk independently. The treatment also resulted in a complete response (CR). Adverse events were minor, enabling the continuation of ICI treatment alongside thyroid hormone and steroid supplementation. Four and a half years later, there has been no recurrence since the initiation of ICI treatment. This case here serves as a reminder that such patients should not be overlooked Table 1.

**Table 1:** Mismatch repair deficient endometrial cancer with PS 3/4 treated with ICI

ICI/	Clinical Outcome	Reference
Dostarlimab (anti-PD-1 Ab) (n = 6)	• Response rate: 100% (CR: 33%) • Median time from PS 3/4 to PS 0/1: 6 weeks • Duration of ICI treatment: 11, 14 months, over 8 months (1 case), over 16 months (2 cases), over 27 months (1 case) • AE: not available • Grade 5: not reported	Ducceschi M [15]
ICI (n = 27, EC: 1 case) MSI-high cancers including CRC (18 cases), GC (5 cases), BC (1 case), PC (1 case), SIC (1 case)	• Response rate: 33% • (EC: Best response: PR) • Improvement from PS 2/3 to PS 0: 52% • Median PFS: 3.4 months • 18-month OS rate: 50.8% (95% CI, 32.7–78.8) • AE (Grade 3): 11% • Fatigue (Grade 1–2): 33% • Pruritus (Grade 1–2): 22% • Hypothyroidism (Grade 1–2): 18% • Mucositis (Grade 1–2): 14% • Diarrhea (Grade 1–2): 7%, (Grade 3): 3% • ALT increased (Grade 3): 3% • AST increased (Grade 3): 3% • Amylase increased (Grade 3): 3% • Lipase increased (Grade 3): 3% • Grade 5: not reported	Pietrantonio F, [16]
Pem (n = 1)	• Best response: PR (almost CR) • Time from PS 4 to PS 0: 10 weeks • Duration of ICI treatment: Over 15 months • AE: hypoadrenalism & colitis (Grade 2)	Watanabe M, [19]
Pem (n = 1)	• Best response: PR (almost CR) • Time from PS 4 to PS 1: 16 months • Duration of ICI treatment: over 57 months • AE: hypoadrenalism & hypothyroidism (Grade 2)	our case

ICI Immune check point inhibitor, Ab Antibody, n Number, Pem Pembrolizumab, PS Performance status, CR Complete response, AE Adverse event, PR Partial response, EC Endometrial cancer, CRC Colorectal cancer, GC Gastric cancer, BC Biliary tract cancer, PC Pancreas cancer, SIC Small intestinal cancer

## Case report

**Fig. 1:**
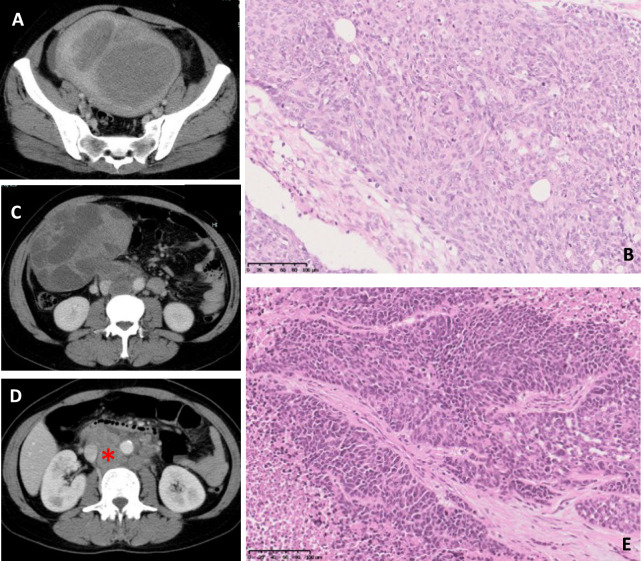
CT and histopathology images **A**. CT scan showing endometrial cancer. **B**. CT scan showing right ovarian metastasis. **C**. Histopathology of endometrial cancer. **D**. CT scan showing PAN metastasis (red asterisk indicates PAN metastasis). **E**. Histopathology of LSLN metastasis

**Fig. 2:**
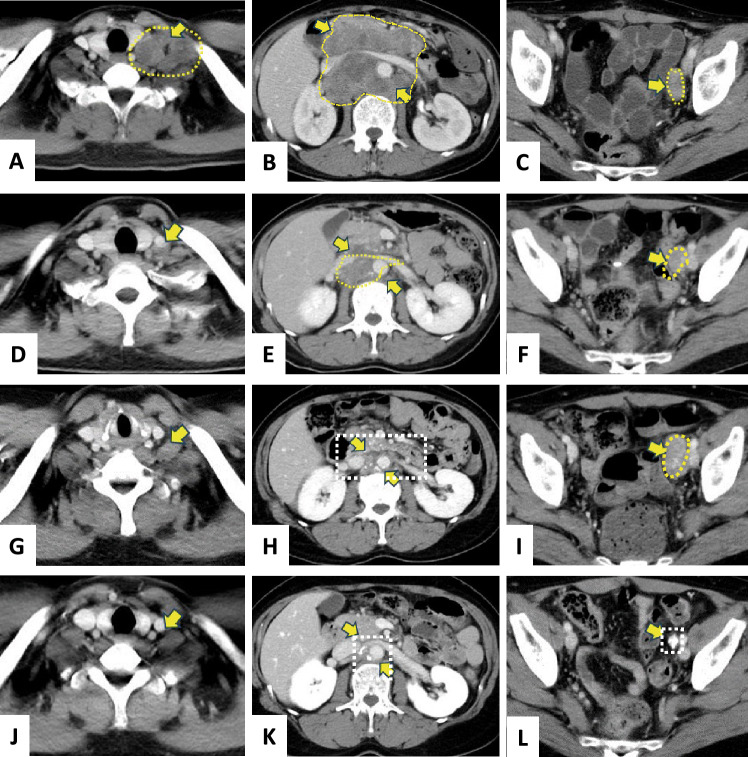
CT scans of target lesions **A**. LSLN metastasis in May 2020. **B**. PAN metastasis in May 2020. **C**. LEIALN metastasis in May 2020. **D**. LSLN metastasis in August 2020. **E**. PAN metastasis in August 2020. **F**. PAN metastasis in August 2020. **G**. LSLN metastasis in January 2021. **H**. PAN metastasis in January 2021. **I**. LEIALN metastasis in January 2021. **J**. LSLN metastasis in May 2024. **K**. PAN metastasis in May 2024. **L**. LEIALN metastasis in May 2024. The yellow arrows and dots mark the lesion area

**Fig. 3:**
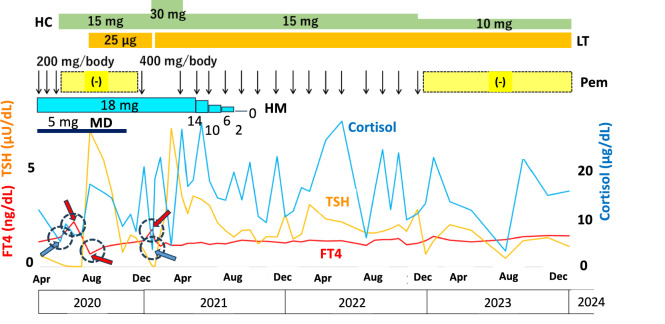
Trends in immune-related adverse events The blue, red, and orange lines show changes in serum cortisol, FT4, and TSH levels, respectively. Blue arrows mark points of Grade 2 hypoadrenalism; red arrows indicate points of destructive thyroiditis and Grade 2 hypothyroidism. HC Hydromorphone, LT Levothyroxine, Pem Pembrolizumab, HM Hydromorphone, MD Methadone

A 46-year-old female patient with no significant medical history discovered a pelvic tumor and underwent a right salpingo-oophorectomy for Grade 3 endometrioid cancer at her previous hospital in November 2019 (Figs. 1A, B). Pathological examination indicated ovarian metastasis from uterine endometrioid cancer, leading to a revised diagnosis of Stage IIIC uterine cancer (Fig. 1C). In January 2020, she had a total hysterectomy and left salpingo-oophorectomy. Following this, she developed enlarged abdominal para-aortic lymph nodes (PAN) (Fig. 1D), and paclitaxel and carboplatin (TC) therapy was initiated in February of the same year. However, a computed tomography (CT) scan in March showed further enlargement of the PAN and metastasis to the left supraclavicular lymph node (LSLN) (Figs. 1E and 2A), indicating that TC therapy was ineffective. Although her previous gynecologist recommended best supportive care, she and her family were not satisfied with this approach and sought a second opinion at our hospital. At that time, she was experiencing severe back pain that hindered her mobility, necessitating a wheelchair for transport. To manage her pain, a previous doctor prescribed hydromorphone (HM) at 18 mg/day in combination with methadone (MD) at 5 mg/day. The rapidly enlarged para-aortic lymph node metastatic tumor displaced the abdominal aorta and inferior vena cava anteriorly by approximately 2 and 4 cm, respectively, from their normal anatomical positions (Fig. 2B). This was thought to be the cause of cancer pain, and her PS was assessed at 4 (Fig. 3). Methadone should be considered when other opioids are ineffective [8]. The patient was already suffering from extremely severe cancer pain that could not be alleviated with opioids at a previous hospital, and in order to rescue this pain, we explored whether there was a method that could have an antitumor effect on the tumor that was causing the cancer pain. This is really a chicken or egg debate. It was reported that immunotherapy was effective for patients with poor PS and locally advanced NSCLC [5]. If there is rationale such as MSI-high that immunotherapy is effective, it should be considered. Laboratory results at her initial visit showed WBC 4200/μL, Neu 3,000/μL, Hb 9.1 g/dL, Plasma 37.1 × 10^3^/μL, Alb 3.6 g/dL, AST/ALT 177/92 U/L, T-Bil 0.3 mg/dL, Cr 0.66 mg/dL, Na/K/Cl 135/4.5/97 mmol/L, CRP 6.65 mg/dL, CEA 4.4 ng/mL, CA19-9 21.6 U/mL, CA125 22.7 U/mL, FT4 1.26 ng/dL, TSH 0.605 μIU/mL, and cortisol 11.50 μg/dL, with no significant abnormalities in major organ functions.

The patient was diagnosed with MSI-high, and in May 2020, treatment with anti-PD-1 antibody Pem at a dose of 200 mg every 3 weeks was initiated (Fig. 3). After three cycles, cortisol levels decreased to 2.5 μg/dL and ACTH levels decreased to 4.1 pg/mL in June 2020 (Fig. 2). Following the suspension of Pem, the patient consulted an endocrinologist at our hospital and was diagnosed with Grade 2 adrenal insufficiency, leading to the administration of hydrocortisone (HC) at 15 mg/day. In July 2020, FT4 levels rose to 1.79 ng/dL while TSH levels fell to 0.038 μIU/mL with no thyroid swelling, no thyroid peroxidase antibody (TPOAb < 9 IU/mL), but with mildly positive thyroglobulin antibody (TgAb 81 IU/mL) and TSH receptor antibody (TRAb 1.2 IU/L). She was followed for suspected destructive thyroiditis. By September, FT4 levels dropped to 0.65 ng/dL and TSH increased to 6.8 μU/mL, and she was diagnosed with Grade 2 thyroid insufficiency, prompting the start of levothyroxine (LT) supplementation at 25 μg/day (Fig. 3). During this period, antitumor effects were noted; however, a CT scan in January 2021 indicated an increase in metastasis to the left external iliac artery lymph node (LEIALN) while Pem was on hold (Fig. 2I). That same month, FT4 and cortisol levels stabilized, and treatment with Pem was resumed at a dose of 400 mg every 6 weeks. In February, adrenal insufficiency and destructive thyroiditis worsened again (cortisol 3.49 μg/dL, ACTH 3.8 pg/mL, FT4 1.89 ng/dL, TSH 0.025 μIU/mL), leading to an increase in HC to 30 mg/day, temporary suspension of LT, and a total of 19 doses of Pem. A CT scan conducted in January 2023 showed continued shrinkage of the left subclavian, mediastinal, and iliac lymph nodes, resulting in a temporary pause of Pem treatment. Fifty months after initiating treatment with Pem, both adrenal insufficiency and destructive thyroiditis were effectively managed with HC and LT. All target lesions had either shrunk or calcified, maintaining a CR as confirmed by CT scans (Figs. 2J–L). The dosages of MD and HM were gradually reduced; MD was no longer needed by December 2020, and HM was discontinued in October 2021 after eight cycles of Pem (Fig. 3). The patient returned to work in May 2021 and is now able to walk independently, with a PS of 1.

Uterine cancer has the highest incidence of MSI-high/dMMR, making MSI/dMMR testing crucial. Additionally, a thorough investigation into the causes of poor PS can reveal instances where ICIs may be beneficial, provided that organ function is preserved. Cases like that of this patient should not be overlooked. It is important to differentiate decreases in PS caused by musculoskeletal disorders or pain during exertion from declines due to other factors, and appropriate treatments should be considered. Furthermore, PS assessment methods and classifications need to be refined.

## Discussion

Endometrial cancer is the most prevalent gynecological cancer in high-income countries, and its incidence is rising globally [9, 10]. In Japan, there were 17,880 new diagnoses in 2019, and 2,644 deaths in 2020, indicating an upward trend [11]. While aging and a reduction in benign hysterectomies contribute to this increase, obesity is the primary risk factor. Obesity complicates both diagnosis and treatment, highlighting the need for further research to establish primary prevention strategies for high-risk women and to enhance survivorship in endometrial cancer patients. Most endometrial cancers are treatable through hysterectomy if detected early, particularly when accompanied by postmenopausal bleeding, but advanced cases tend to have a poor prognosis [9].

Treatment decisions are influenced by the lesion’s location, the patient’s PS, comorbidities, and the presence of hormone receptors [12]. The preferred drug therapies include TC therapy or doxorubicin combined with cisplatin; however, options become limited once these treatments fail [12]. The incidence of MSI-high in endometrial cancer is approximately 17%, which is the highest among cancers [7]. Furthermore, it has been reported that endometrial cancer with MSI-high/dMMR constitutes about 25–31% of all cases [13].

Compared to microsatellite-stable endometrial cancers, MSI-high/dMMR endometrial cancers exhibit a higher neoantigen burden and contain greater numbers of CD3-positive, CD8-positive, PD-1-expressing tumor-infiltrating lymphocytes, and PD-L1-expressing immune cells within and around the tumor [14].

The anti-PD-1 antibody Pem demonstrates antitumor activity in patients with MSI-high/dMMR endometrial cancer [7]. In the KEYNOTE-158 study, Pem provided sustained antitumor effects and favorable survival outcomes, with manageable toxicity in patients with advanced MSI-high/dMMR endometrial cancer who had previously received treatment and had a PS of 0–1 [7]. Additionally, about 25–31% of endometrial cancers are reported to be MSI-high or dMMR [13]. However, the efficacy and safety of ICIs for advanced endometrial cancer with poor PS have not yet been fully validated.

With the same PS grade, patients may have varying conditions. In this case, pain from a large tumor reduced ADL, worsening PS.

Although the apparent PS was low, major organ function remained intact. PS assessment is subjective and may be affected by factors such as cancer pain and ADL impairment from bone metastases, which can lower the observed PS.

Lee et al.’s recent analysis identified a significant inverse relationship between low albumin levels and progression-free survival (PFS) following ICI therapy [15]. Alongside poor PS and hypoalbuminemia, liver metastases were also notably linked with lower survival rates in NSCLC patients treated with ICIs. Additionally, examining individual metastases in ICI responders indicated a generally uniform response across distant lesions, suggesting that systemic immune responses mediated by peripheral cloned T cells might exert a greater effect on antitumor responses than locally infiltrating lymphocytes [16].

Although molecular and genomic testing holds shows potential for predicting outcomes, it cannot fully replace the critical roles of nutritional status, functional reserve, and metastatic disease in decisions about treatment continuation. Current predictive tools for ICI efficacy are limited; thus, a more detailed combination of patient characteristics and molecular markers is needed to optimize future treatments [1].

While ICIs have proven effective and safe in NSCLC, the impact on patients with poor PS remains less defined. Real-world data in lung cancer showed that 101 (80.8%) out of 125 patients had an ECOG PS of ≥ 2. Within 12 months, 50 immune-related adverse events (irAEs) were observed, but patient factors did not significantly affect irAE occurrence or overall survival (OS), except for race (p = 0.045) [17].

Tomasik et al. conducted a systematic review and meta-analysis across 67 interventional and observational studies (26,442 patients) to evaluate the efficacy and safety of ICIs in NSCLC patients with PS ≤ 1 versus PS ≥ 2 [18]. Patients with a lower PS were approximately twice as likely to have reduced responsiveness to ICIs compared to those with PS ≤ 1, indicating that a lower PS is both prognostic and predictive of ICI response, though it did not affect the safety profile. The pooled hazard ratio for PFS was 2.17 (95%CI 1.96–2.39; I2, 65%), and for OS, it was 2.76 (95%CI, 2.43–3.14; I2, 76%). However, the odds ratio for adverse events (AEs) was 1.12 (95%CI 0.84–1.48), showing a consistent safety profile regardless of PS. Prospective randomized studies are still needed to establish the potential benefits of ICIs in patients with low PS.

In the KEYNOTE-158 study, Pem showed an objective response rate of 48% in patients with MSI-high/dMMR advanced endometrial cancer, with a median response duration of 48 months, median PFS of 13.1 months, and median OS of at least 48 months [7]. Pem’s toxicity was manageable, with most treatment-related AEs being mild to moderate in severity among the 90 patients evaluated for safety. Treatment discontinuations due to treatment-related or immune-mediated AEs were low, affecting six and two patients, respectively [7]. However, the study included only patients with PS 0–1, excluding those with advanced endometrial cancer and poor PS from the trial.

Multiple reports indicate that ICIs can be highly effective with manageable toxicity in advanced endometrial cancer patients with poor PS. Ducceschi et al. documented a multicenter case series of six patients with PS 3–4 treated with the anti-PD-1 antibody costalimai for advanced endometrial cancer, showing rapid responses and notable PS improvement [15]. The median time for clinical response (from PS 3–4 to PS 0–1) was 6 weeks. In these cases, PS 3 or higher was solely attributed to disease burden in 5 out of 6 cases. In these cases, cancer pain decreased PS. Pietrantonio et al. also demonsrtated anti-PD-1 antibody efficacy in 27 patients with MSI-high/dMMR malignancies and PS 2 (74%) or PS 3 (26%), including one endometrial cancer case. After a median of 6 weeks, 52% improved to PS 1, and of these, 30% further improved to PS 0 after 10 weeks. Grade 3 or higher AEs occurred in 11% (3/27) of cases, including colitis, hepatitis, and pancreatitis, with no treatment-related deaths reported. In the present case, disease control lasted over 48 months, with PS improving from 4 to 0. Grade 2 adrenal insufficiency and hypothyroidism were managed by temporarily suspending Pem and using appropriate hormone replacement. Watanabe et al. reported a case involving a young woman with a PS of 4, primarily due to cancer pain from tumor invasion into the pelvic wall, but with intact cardiac, hepatic, and renal function, no comorbidities, and no prior medical history. Treated with Pem alone, her PS improved to 0 within 10 weeks [19]. In this case, her poor PS was solely disease-related, without any organ dysfunction. Such occurrences are termed Lazarus-type responses, named after the biblical resurrection story [20]. These reactions are commonly observed in NSCLC and, to a lesser extent, in endometrial cancer. Although the precise mechanisms remain unclear, attention to this in phenomenon in MSI-high/dMMR endometrial cancer is advised. Furthermore, assessing whether poor PS in advanced endometrial cancer stems from the disease itself, other complications, or organ failure may be crucial when considering Pem therapy.

## Conclusion

This case demonstrates the high efficacy of an ICI in a patient with MSI-high/dMMR advanced endometrial cancer and a PS of 4, initially deemed suitable only for supportive care, achieving long-term survival. When poor PS results from disease burden alone, without additional complications or organ failure, Pem may enhance both survival and PS with manageable toxicity.

## Supplementary Information

Below is the link to the electronic supplementary material.

Supplementary file1 (PDF 843 KB)

## Data Availability

Raw data were generated at Akita university. Derived data supporting the findings of this study are available from the corresponding author HS on request.
