# Supplementary figures and images for: Immune checkpoint inhibitor restores daily function in patient with microsatellite instability (MSI)-high advanced endometrial cancer and poor performance status
Source: Int Cancer Conf J. 2025 Feb 14;14(2):163–70. doi: 10.1007/s13691-025-00752-3 (PMC11950546; doi:10.1007/s13691-025-00752-3)

Supplemental Fig. 1

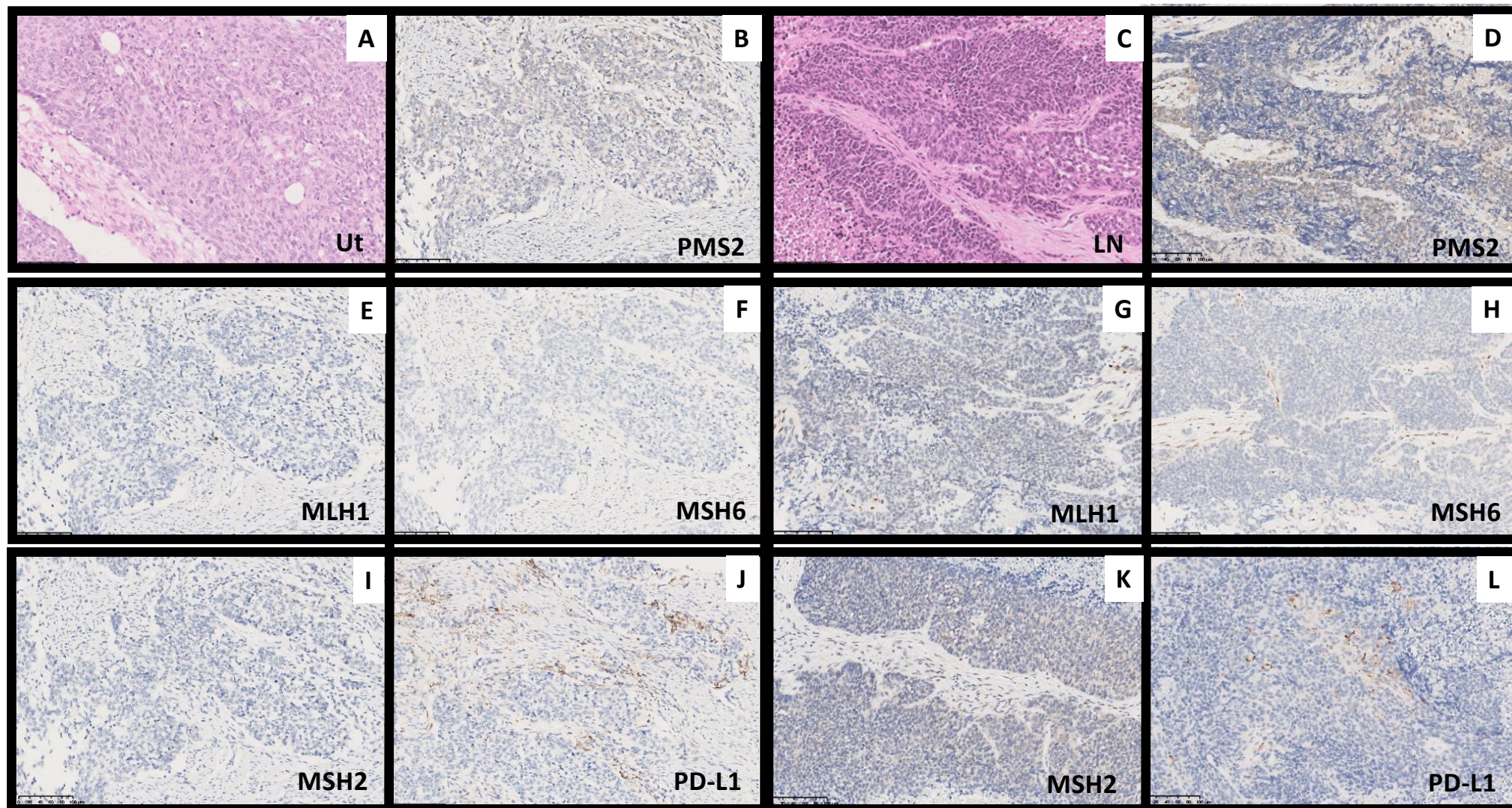

Supplement: Supplementary file 1 — Supplementary file1 (PDF 843 KB) [file 13691_2025_752_MOESM1_ESM.pdf]
